# 3D Artificial Skin Platform for Investigating Pregnancy-Related Skin Pigmentation

**DOI:** 10.3390/mi15040511

**Published:** 2024-04-10

**Authors:** Uiechan Jeong, Sunhee Yoon, Sungjin Park, Tae-Joon Jeon, Sun Min Kim

**Affiliations:** 1 Department of Mechanical Engineering, Inha University, 100 Inha-ro, Incheon 22212, Republic of Korea; uchanj@gmail.com; 2 Department of Biological Sciences and Bioengineering, Inha University, 100 Inha-ro, Incheon 22212, Republic of Korea; yoonsh0912@inha.ac.kr; 3 Department of Mechanical and System Design Engineering, Hongik University, 94 Wausan-ro, Seoul 04066, Republic of Korea; parksj@hongik.ac.kr; 4 Department of Biological Engineering, Inha University, 100 Inha-ro, Incheon 22212, Republic of Korea

**Keywords:** artificial skin platform, 3D artificial skin, pregnancy-related skin pigmentation, oxygen concentration, 17β-Estradiol, melanin expression

## Abstract

In this study, we created a 3D Artificial Skin Platform that can be used for the treatment of pigmentation by artificially realizing the skin of pregnant women. For the stable realization of 3D artificial skin, a bilayer hydrogel composed of collagen type I and fibrin was designed and applied to the study to reduce the tension-induced contraction of collagen type I, the extracellular matrix (ECM) of artificial skin, by dynamic culture. Oxygen concentration and 17β-Estradiol (E2) concentration, which are highly related to melanin production, were selected as parameters of the pregnancy environment and applied to cell culture. Oxygen concentration, which is locally reduced in the first trimester (2.5–3%), and E2, which is upregulated in the third trimester, were applied to the cell culture process. We analyzed whether the 3D artificial skin implemented in the 3D Artificial Skin Platform could better represent the tendency of melanin expression in pregnant women than cells cultured under the same conditions in 2D. The expression levels of melanin and melanin-related genes in the 2D cell culture did not show a significant trend that was similar to the melanin expression trend in pregnant women. However, the 3D artificial skin platform showed a significant trend towards a 2-6-fold increase in melanin expression in response to low oxygen concentrations (2.5%) and E2 concentrations (17 ng/mL), which was similar to the trend in pregnant women in vivo. These results suggest that 3D artificial skin cultured on the Artificial Skin Platform has the potential to be used as a substitute for human pregnant skin in various research fields related to the treatment of pigmentation.

## 1. Introduction

A woman’s body undergoes changes in hormones and oxygen levels during pregnancy [1,2]. These changes create a more favorable environment for the production of melanin in the body, which is then upregulated [3]. This upregulation of melanin expression can cause excess melanin to be produced in the skin, resulting in skin pigmentation, which is when the skin becomes darker than the surrounding skin color. Skin pigmentation is a very common condition in pregnant women, with about 90% of pregnant women reported to have it during pregnancy [4]. Melasma, one of the most common pigmentations, is often referred to as the “mask of pregnancy” and has been reported in various studies as a common problem that affects more than half of pregnant women on average [5,6].

Skin pigmentation is thought to be caused by a variety of internal and external factors, including hormonal changes, inflammation, injury, and acne, which, in combination, are thought to upregulate melanin, leading to disease [7]. However, the detailed mechanisms of the darkening process are still not fully understood.

Skin pigmentation is usually not directly related to health because it is a disease that changes the color of the skin. However, this change in appearance can ultimately have a negative impact on a pregnant woman’s emotional and psychological health. In fact, pregnant women with Chloasma Melasma (CM) experience a lower quality of life compared to those without the condition [6]. As a result, there is a growing demand for skin pigmentation treatment among the significant number of pregnant women who suffer from pigmentation [4]. For example, hydroquinone is the most commonly used treatment for melasma but is classified by the U.S. Food and Drug Administration (U.S. FDA) as C level within the pregnancy category. The U.S. FDA pregnancy category C is reserved for drugs that have been shown to have adverse effects on the fetus in animal studies, but not enough studies have been done in humans. It is therefore difficult to apply to pregnant women in general, so it is necessary to find measures and treatments for skin pigmentation that can be used in the specific situation of pregnancy [8].

Regarding skin pigmentation, the main factor that gives skin its color is melanin. Melanin is expressed in melanocytes and gives skin, hair, and eyes their unique color. The color that melanin gives to the body is determined by the amount of melanin and the type of melanocytes that express it. The main elements of melanin that produce these colors are eumelanin, which is responsible for darker colors, and pheomelanin, which is responsible for lighter colors [9]. Melanin is also believed to be involved in wound healing and immune response, as well as protecting the skin and underlying tissues from the harmful effects of ultraviolet (UV) light [10,11].

To explain how melanocytes give skin its color, melanocytes produce organelles called melanosomes in their nuclei. As these melanosomes mature inside the melanocyte, they are transported to the cell’s peripheral border and, through several transport mechanisms, are transferred from the dendritic tip of the melanocyte to the surrounding keratinocytes, where they give the skin its color [11].

While melanin has a clear role in the body, it can also cause skin pigmentation when it is overproduced. There are many ways to treat melanin-induced pigmentation, including topicals, medications, and laser treatments, but identifying the cause and choosing the appropriate treatment is key to the effective treatment of skin pigmentation [12]. Skin pigmentation in pregnant women is caused by a variety of complex factors, so there is no single cause. Treatment without identifying the cause is not only ineffective, but also inappropriate [13].

In this study, we developed an Artificial Skin Platform for the treatment of skin pigmentation in pregnant women. Commonly used in cell and tissue research, 2D cultures allow for simple, rapid culture of cells and have a low maintenance cost. However, the lack of cell-to-cell interaction in 2D culture can negatively affect cell differentiation, proliferation, and gene expression. It has also been reported that the morphology of cells cultured in 2D cell culture plates tends to appear relaxed and stretched, unlike cells in their natural state [14,15,16]. In other words, these limitations make biomimicry in 2D cell culture very limited. Further, 3D cultures can overcome many of the limitations of 2D cultures in terms of organ modeling.

The developed Artificial Skin Platform provides cells with a microenvironment with a spatial structure similar to that of real organs, making cell-cell and cell-environment interactions easier than in 2D. The similarity to organs in these 3D culture environments is higher than when cells are cultured in 2D, so they exhibit similar mechanisms to those seen in the real body [14,15,16,17,18].

**Figure 1:**
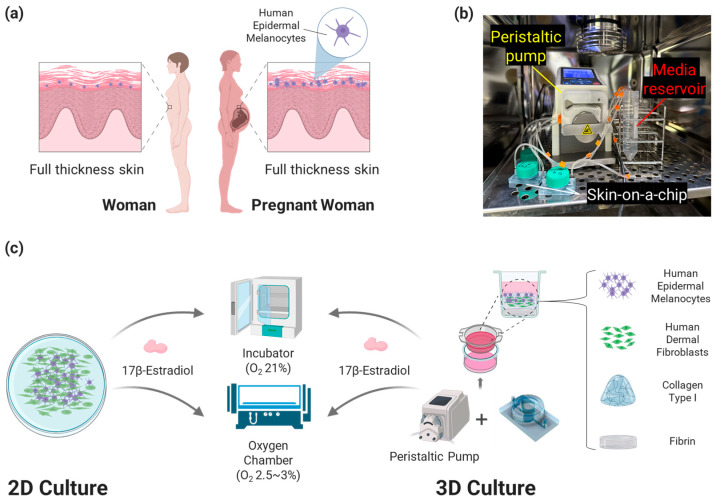
Research background, experimental setting, and schematic figure of the research. (**a**) Comparison of the skin of pregnant and non-pregnant women. In general, pregnant women have more pigmentation than non-pregnant women, due to the overexpression of melanocytes with various causes. (**b**) Dynamic culture setting with skin-on-a-chip and pump in the incubator. (**c**) Overall scheme of the study. We demonstrated that melanin, located in the basal layer of the skin’s epidermis, is upregulated by the changing body environment when a woman becomes pregnant, causing pigmentation in the form of 3D artificial skin. To realize 3D artificial skin, cells were seeded inside and outside the bilayer hydrogel and inserted into the Artificial Skin Platform for dynamic culture. Comparative analysis of the 3D artificial skin cultured on the Artificial Skin Platform with the 2D static cultured cells under the same culture conditions showed that the melanin expression tendency of the 3D artificial skin was more similar to that of pregnant women than the 2D cultured cells.

We designed a bilayer hydrogel composed of collagen type I and fibrin for 3D cell culture (Figure 1). This is a new type of Skin Platform to prevent excessive shrinkage of collagen type I, which is widely used as the ECM of 3D artificial skin. Human Dermal Fibroblasts (HDF) were seeded into collagen type I to create a dermis layer, and Human Epidermal Melanocytes (HEM) were seeded on top of it and cultured to create 3D artificial skin. To analyze the degree of pigmentation due to changes in the environment of the pregnant woman’s body, oxygen and E2 concentrations, which are related to melanin production and change rapidly in the pregnant woman’s body throughout pregnancy, were applied to the cell culture. In addition, when culturing 3D artificial skin, the Artificial Skin Platform was connected to a peristaltic pump to perform dynamic culture at a flow rate of 1 μL/min. Compared to static culture, dynamic culture is suitable for cell growth and can simulate blood flow, so it was applied to the developed platform [19,20]. The flow rate was determined based on research on ECM and cells in 3D artificial skin [21].

The first-trimester oxygen concentration and third-trimester oxygen and E2 concentrations of pregnant women were applied to 3D artificial skin cultures on the Artificial Skin Platform to analyze the degree of melanin production [1,22,23,24,25,26,27,28,29,30]. To investigate the high biomimicry of the platform, cells were cultured in 2D in the same environment and the expression of melanin and melanin-related genes were compared with the melanin expression trend of 3D artificial skin. We found that the expression of melanin in 3D artificial skin was more similar to that of pregnant women in vivo than in 2D culture, confirming that the Artificial Skin Platform has the potential to be used in various research fields related to the treatment of skin pigmentation in pregnant women.

## 2. Materials and Methods

### 2.1. Skin-on-a-Chip

Skin-on-a-chip utilizes a design that has been widely used for 3D culture in previous studies [31,32]. Transwell 24-well cell culture plates (24 Transwell; SPL Life Sciences, Cat. No. 36124, Pocheon, Republic of Korea) were set up as culture wells and inserted into the chip to perform dynamic culture.

**Figure 2:**
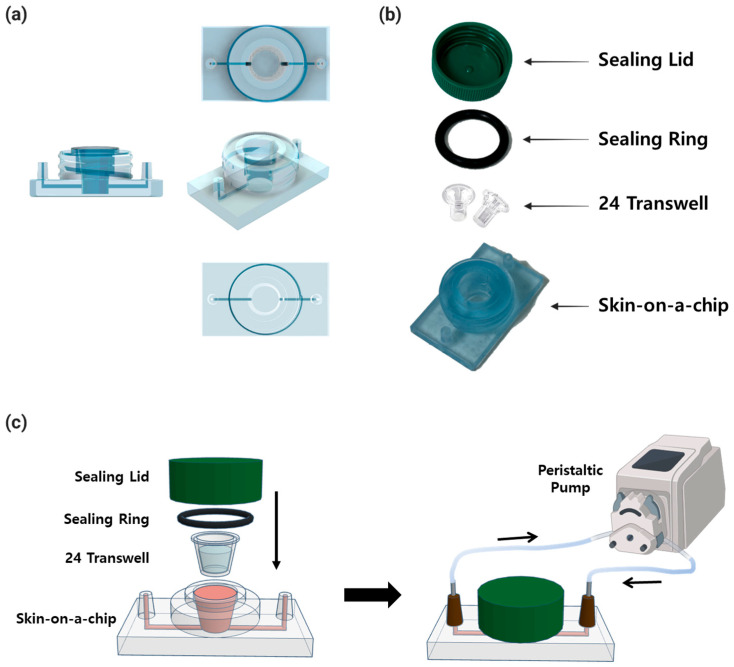
Overall configuration and morphology of the skin-on-a-chip fabricated in this study. (**a**) View of the skin-on-a-chip design from different angles. (**b**) Fabricated chip and all components required for 3D artificial skin culture on the skin-on-a-chip. (**c**) The schematic figure of chip combinations and operation steps.

The system consisted of a skin-on-a-chip, a 20.0 × 3.5 mm sealing ring (O-Ring-STOCKS, Cat. No. 190539, Wezep, The Netherlands), a sealing lid utilizing a 50 mL conical tube (SPL Life Sciences, Cat. No. 50150, Pocheon, Republic of Korea), and 24 Transwells with 3 μm pores (Figure 2). The skin-on-a-chip, which is 50 mm across and 30 mm long, has one inlet and one outlet with a diameter of 4 mm at each end, and the length of the channel between them is 41 mm. In the center is a well for inserting 24 Transwells, with a diameter of 11 mm and a height of 13 mm. The chip was fabricated with a non-toxic resin for 3D printers (Carima, Cat. No. 5097979637, Seoul, Republic of Korea) on a 3D printer (Figure 2a). The sealing ring, lid, Transwell, and chip were combined and sealed. After the combination, the whole skin-on-a-chip was connected to a peristaltic pump to culture (Figure 2b,c).

### 2.2. Cell Culture

HDF (ScienCell Research Laboratories, Cat. No. 2310, Carlsbad, CA, USA) used cells between passages 4 and 6. Culture media was Fibroblasts Media (FM; ScienCell Research Laboratories, Cat. No. 2301, Carlsbad, CA, USA) containing 5% Fetal Bovine Serum (FBS; ScienCell Research Laboratories, Cat. No. 0010, Carlsbad, CA, USA), 1% Penicillin/Streptomycin (P/S; ScienCell Research Laboratories, Cat. No. 0503, Carlsbad, CA, USA), and 1% Fibroblast Growth Supplement (FGS; ScienCell Research Laboratories, Cat. No. 2352, Carlsbad, CA, USA). HEM (ScienCell Research Laboratories, Cat. No. 2200, Carlsbad, CA, USA) used cells between passages 4 and 7. Culture media was Melanocytes Media (MelM; ScienCell Research Laboratories, Cat. No. 2201, Carlsbad, CA, USA) containing 1% Melanocyte Growth Supplement (MelGS; ScienCell Research Laboratories, Cat. No. 2252, Carlsbad, CA, USA), 1% P/S, 0.5% FBS. Also, 0.25% Trypsin-EDTA (Gibco, Cat. No. 15400, Grand Island, NY, USA) was used to harvest the cells when the cells reached 80–90% confluency.

### 2.3. Oxygen and Hormone Level Control

To create a hypoxic environment in the incubator, a hypoxia chamber (BioSpherix, model C-274, Redfield, NY, USA) and a ProOx 110 oxygen controller (BioSpherix, P110, Redfield, NY, USA) were used. The oxygen concentration in the hypoxia chamber was set to 2.5–3% to reflect the hypoxic environment of a pregnant woman in the first trimester [1,22,23,24,25].

E2 (Cerilliant, Cat. No. E-060-1ML, Round Rock, TX, USA) was diluted in the media to mimic the hormone in pregnant women. E2 dilution concentrations were determined based on the average E2 concentration in non-pregnant women and the E2 concentration in the third trimester of pregnant women. The concentrations of E2 used in the experiments were 0.3 ng/mL, 7 ng/mL, and 17 ng/mL [26,27,28,29,30].

### 2.4. Two-Layer Hydrogel Shrinkage Test

Collagen type I (Corning, Cat. No. 354249, New York, NY, USA), Fibrinogen (Sigma-Aldrich, Cat. No. 9001-32-5, St. Louis, MO, USA) and thrombin (Sigma-Aldrich, Cat. No. 9002-04-4, St. Louis, MO, USA) were used as an extracellular matrix for the experiments. In order to minimize structural deformation of the extracellular matrix during long-term culture, we employed a strategy of using a fibrin layer, known for its minimal contraction properties, at the base of the collagen type I layer. This approach effectively counteracts the inherent tendency of collagen type 1 to undergo significant contraction when in direct contact with the cell culture media [33,34,35,36].

Fibrin was produced by mixing fibrinogen and thrombin in a 1:1 ratio of fibrinogen 10 mg/mL and thrombin 10 U, which exhibited a dilution-dependent elution and no morphology change under dynamic flow of the fluid [37]. The concentration of collagen type I was set to 6 mg/mL, which is appropriate for the ECM of 3D artificial skin [38]. To verify the effect of fibrin, three types of hydrogels were tested: (i) fibrin (100 μL) and collagen type I (500 μL) with 1 μL/min dynamic flow of media, (ii) collagen type I (500 μL) with 1 μL/min dynamic flow of media, and (iii) collagen type I (500 μL) with static condition. Since the hydrogel showed a change in height but not area as the experiment continued, the height was measured with a vernier caliper every 24 h. The height of the hydrogel was measured only in the collagen type I layer.

### 2.5. Hematoxylin and Eosin Staining

Hematoxylin and eosin (H & E) staining was performed to identify the nuclei and cytoplasm of the tissue [39]. To stain the tissues, the cultured tissues were prepared into paraffin blocks. The prepared paraffin blocks were cut into 10 μm-thick sections using a Microtome (Leica Biosystems, RM2165, Nussloch, Germany), and the sections were deparaffinized and placed on slides. The tissues were treated with Hematoxylin (Modified Mayer’s Solution) and Eosin Y solution from the H & E Staining Kit (Abcam, Cat. No. ab245880, Cambridge, UK). The tissue was washed with absolute alcohol (Sigma-Aldrich, Cat. No. 64-17-5, St. Louis, MO, USA), and the stained tissue was analyzed under a confocal microscope.

### 2.6. Fontana Mason Staining

To identify the melanin produced by cells in the tissue, we performed a Fontana Mason stain, which stains specifically for melanin [40]. For Fontana Mason staining, the cultured 3D artificial skin was first prepared as a paraffin block and cut into 10 μm-thick sections using a Microtome. The cut paraffin block sections were stained according to the protocol of the Fontana Masson stain kit (Abcam, Cat. No. ab150669, Cambridge, UK). The samples were analyzed for stained melanin under a confocal microscope.

### 2.7. Quantitative Real-Time PCR (qRT-PCR)

To analyze the expression of melanin-related genes, RT-PCR was performed. RNA was extracted from the cells using the RNeasy Mini Kit (QIAGEN, 74104, Valencia, Spain). For RT-PCR analysis of the extracted RNA, the extracted RNA was synthesized into cDNA by following the protocol of the Primescript™ RT Master Mix (Takara Bio Inc., RR036, Kusatsu, Japan) kit. The primers designed to detect the target gene were mixed with the TB Green^®^ Premix Ex Taq™ II (Takara Bio Inc., RR820 Kusatsu, Japan) kit to performed RT-PCR. RT-PCR analysis was performed using the CFX Opus 96 Real-Time PCR System (Bio-Rad Laboratories, Hercules, CA, USA) according to the protocol of TB Green^®^ Premix Ex Taq™ II. The primers used in this experiment are shown in Table 1.

### 2.8. Quantitative Melanin Analysis

A melanin quantification method based on absorbance measurements was used in this study [41]. A 24-well cell culture plate was seeded with 100 μL of HDF diluted to a concentration of 1 × 10^6^ cells/mL. Then, 400 μL of FM was added to each well seeded with cells and incubated in the incubator for 1 day. HEM, diluted to a concentration of 1 × 10^6^ cells/mL, was seeded 100 μL into the wells seeded with HDF. A co-culture medium consisting of FM and MelM mixed in a 1:1 volume ratio was used for the co-culture of the two cells. Co-culture medium containing 0.3, 7, and 17 ng/mL of E2, respectively, was added to each well for each experimental condition. Half of the cell-seeded well plates were incubated in an incubator, and the other half were incubated in a hypoxia chamber inside the incubator, where the oxygen concentration was adjusted to 2.5–3% using an Oxygen Controller. Each well was incubated for 3, 6, and 9 days, and the culture medium was changed every other day.

After incubation, the wells were washed twice with DPBS. Then, 250 μL of 1 M NaOH was added to each well and incubated for 1 h in an incubator to lysate the cells. The lysed solution was transferred to a 96-well plate (SPL Life Sciences, Cat. No. 30096, Pocheon, Republic of Korea) in 100 μL increments and analyzed for melanin content by measuring absorbance at a wavelength of 405 nm with a Plate Reader (Thermo Fisher Scientific, 3020-141, Waltham, MA, USA).

### 2.9. 3D Artificial Skin Culture on the Artificial Skin Platform

**Figure 3:**
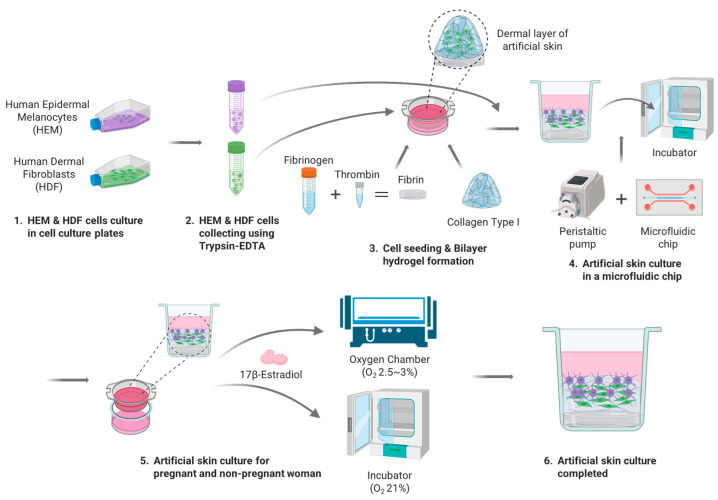
Protocol for 3D artificial skin culture on the Artificial Skin Platform for the treatment of skin pigmentation in pregnant women: 1. HDF and HEM cells culture in cell culture plates; 2. HDF and HEM cells collected using Trypsin-EDTA; 3. Cell seeding and bilayer hydrogel fabrication; 4. 3D artificial skin culture in a microfluidic chip; 5. 3D artificial skin culture for pregnant and non-pregnant women; 6. 3D artificial skin culture completed.

The 3D artificial skin implemented by the Artificial Skin Platform for the treatment of skin pigmentation in pregnant women was cultured as follows (Figure 3). HDFs and HEMs used for the 3D artificial skin were cultured as described previously. The seeding concentration of HDF, HEM was 1 × 10^6^ cells/mL. To realize the 3D artificial skin, the previously described bilayer hydrogel was implemented in 24 Transwells. At this time, 400 μL of Collagen Type I, which is responsible for the ECM of the dermis, and 100 μL of HDF at a concentration of 1 × 10^6^ cells/mL were diluted to adjust the final concentration of collagen type I to 5 mg/mL, seeded into 24 Transwells, and gelated for 30 min in an incubator. Once the gelation of collagen type I was complete, FM mixed with 5% FBS, 1% P/S, 1% FGS for HDF culture was added to the wells and immersion cultured for one day. Upon completion of the one-day immersion culture, the media administered in the wells was removed and 100 μL of HEM at a concentration of 1 × 10^6^ cells/mL was seeded, and a 1:1 mixture of FM with 5% FBS, 1% P/S, 1% FGS and MelM with 1% MelGS, 1% P/S, 0.5% FBS was administered to the wells for two days of immersion culture. After two days of immersion culture stabilized the adhesion of the seeded cells, 24 Transwells were inserted into the wells in the middle of the skin-on-a-chip and connected to the peristaltic pump. The flow rate of the peristaltic pump was set to 1 μL/min and the cells were cultured for 7 days, replacing half of the total media with fresh media every 2 days. For the analysis of melanin expression in pregnant and non-pregnant women, different oxygen concentrations and E2 were applied to each of the six experimental groups. Three experimental groups were cultured in a hypoxia chamber with oxygen concentration set at 2.5–3%, with 1:1 dilution media of FM and MelM diluted to E2 concentrations of 0.3, 7, and 17 ng/mL, respectively, fed into and out of the wells every other day. The remaining three experimental groups were cultured with an oxygen concentration of 21% with 1:1 diluted media of FM and MelM diluted to E2 concentrations of 0.3, 7, and 17 ng/mL, respectively, fed inside and outside the wells every other day. The cultured 3D artificial skin was analyzed by H & E staining and Fontana Masson staining, as described in Section 2.

## 3. Results and Discussion

### 3.1. Shrinkage of a Bilayer Hydrogel

Collagen is the most abundant ECM substance in the skin and is commonly used in artificial skin research [42]. Fibrin is an insoluble protein formed by the action of thrombin on fibrinogen in plasma and is used as the ECM in artificial skin experiments [43]. The non-shrinking nature of fibrin in fluids is advantageous for histological analysis, as it allows tissues to remain intact in long-term culture [36]. As you can see, each hydrogel has its own unique properties, and determining the right hydrogel for your experiment is an important aspect of artificial skin research.

The ECM for this experiment was collagen type I, which makes up three-quarters of the weight of human skin, excluding water, and provides a 3D scaffolding with excellent cell and tissue compatibility [44]. However, collagen type I may not be suitable for long-term culture due to its high biodegradability and weak cross-linking, which can easily cause the hydrogel to shrink [35]. In other words, reducing the degree of shrinkage of the collagen type I hydrogel to maintain tissue shape is important in biomimicry.

In long-term culture studies of artificial skin, there are several factors that can affect collagen type I hydrogel shrinkage: cellular, dehydration, and tension [33,34,35]. Seeding and culturing HDF within collagen type I hydrogel is essential in terms of realizing the dermis of a 3D artificial skin. Therefore, we have not considered the hydrogel shrinkage caused by culturing cells within the hydrogel, but have considered other factors that cause shrinkage. In terms of simulating the pigmented skin of pregnant women, dynamic flow in cell culture is essential to simulate the blood flow that supplies nutrients to the skin. When dynamic flow is applied to the culture, the continuous tension applied to the lower layer of the hydrogel composed of collagen type I and the dehydration of the hydrogel due to temperature changes in the fluid can cause hydrogel shrinkage by shrinking collagen fibrils or increasing the spacing between collagen molecules [34]. In this study, we designed a bilayer structure of fibrin and collagen type I and performed experiments with the aim of reducing the contraction of collagen type I due to fluid tension by preventing the direct contact of fibrin with collagen type I and dynamic flow.

**Figure 4:**
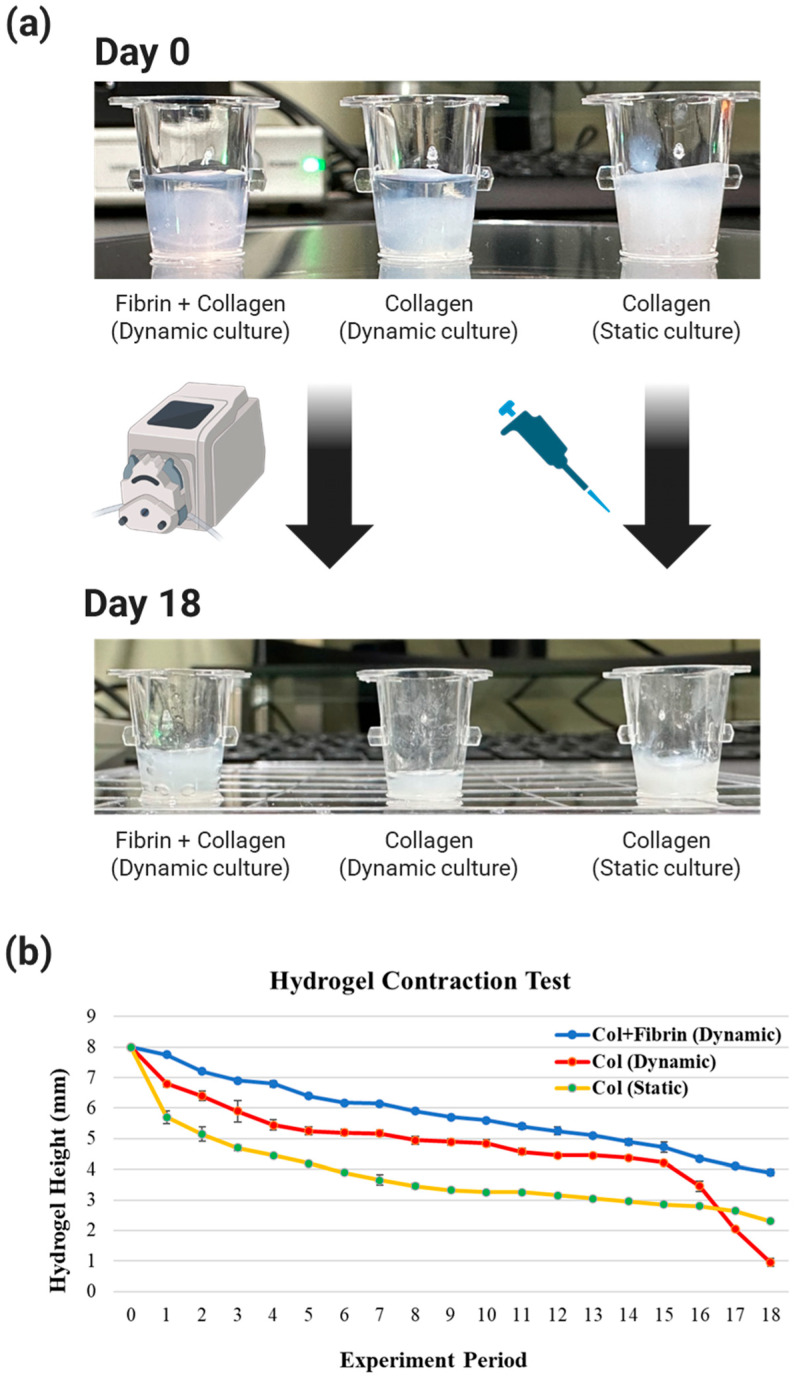
Evaluation of shrinkage of bilayer hydrogel of fibrin and Collagen Type I. (**a**) Comparison of hydrogel on day 0 and day 18 of three experimental groups. (**b**) The height of the hydrogel of the three experimental groups was measured and plotted for a total of 18 days. Col indicates collagen type I. Also, Dynamic means dynamic culture, and Static means static culture.

The three experimental groups were divided Into three experimental groups according to the type of hydrogel and media used in the experiment. The degree of shrinkage of each experimental group was visibly different at day 0 and day 18, depending on the type of hydrogel and media (Figure 4a).

The measurements showed that the most significant changes in each group occurred at two time points: days 1–3 and days 15–18 (Figure 4b). Rapid hydrogel shrinkage occurred in all experimental groups on days 1–3. Since the initially seeded collagen type I hydrogel contains a lot of water, it is expected that the shrinkage is caused by the escape of the contained water in response to the relatively high temperature of 37 °C and the tension caused by the fluid.

Overall, the bilayer structure of fibrin and collagen type I showed very low shrinkage compared to the other experimental groups, and the static culture of collagen type I showed higher shrinkage compared to the dynamic culture. This is due to the fact that in a static culture with a small volume of media at 37 °C, the temperature of the media quickly increases. This means that the temperature of the collagen increases, and since collagen shrinks proportionally with temperature, it is believed that the shrinkage is higher than in the dynamic culture. Since the media is continuously fed into the hydrogel, the temperature of the media is lower than that of the media in the static culture, and therefore the degree of shrinkage seems to be less. This suggests that the incubator temperature has a greater effect on hydrogel shrinkage than the tension-induced shrinkage caused by the flow rate of 1 mL/min.

The change from day 15 to day 18 was a significant shrinkage of collagen (dynamic culture), whereas the shrinkage of the other experimental groups was not significant. Collagen (dynamic culture) was subjected to shear stress from the flowing fluid and a decrease in adhesion strength due to hydrogel shrinkage, causing the hydrogel to fall off the contact surface with the 24 Transwells. After that, collagen (dynamic culture) was subjected to tension from the flowing fluid in all directions, causing the hydrogel to shrink rapidly to about 1/3 of the height of collagen (static culture) on day 18. This phenomenon of collagen (dynamic culture) hydrogel is a common occurrence in long-term cultures that are constantly exposed to flowing fluids. Therefore, the use of collagen (dynamic culture) in experiments has been shown to be detrimental to the reproducibility and efficiency of experiments.

In other words, the hydrogel of fibrin+collagen (dynamic culture), which is composed of collagen type I, a component that accounts for 3/4 of the weight of dynamic culture and real skin, which has less shrinkage of the hydrogel compared to other experimental groups and is favorable for cell growth, was determined to be the ECM of the artificial skin in this study.

### 3.2. Pigmentation of 2D Artificial Skin Based on the Environment of a Pregnant Woman’s Body

In order to emulate the pigmentation that occurs on a pregnant woman’s skin during pregnancy, we needed to simulate the environment of a pregnant woman’s body. To do so, we chose oxygen levels and hormone levels, which are associated with melanogenesis and change dramatically during pregnancy, as independent variables in the experiment. During the first trimester of a woman’s pregnancy, extravillous trophoblast (EVT) cells invade the decidua, causing a blockage of the uterine spiral arterioles. This prevents maternal blood flow into the intrauterine space and creates a physiologic hypoxic environment [1,22,23,24]. Accordingly, the oxygen concentration of the decidua is 5–6% on average, the chorionic region is 2–3% in the first trimester, and the placenta is 2–3% at 7–9 weeks of pregnancy. In this study, we set the oxygen concentration of the experiment to 2.5–3%, the lowest hypoxic zone, to clearly see the difference in melanin expression according to the oxygen concentration [22,25].

E2 is a type of estrogen hormone that plays a major role in the development and maintenance of female reproductive or secondary sex characteristics. E2 was chosen for this experiment because it is known to be involved in the production of melanin [45,46]. The E2 concentration in this experiment was determined based on research showing that pigmentation is activated in the third trimester in pregnant women [47,48,49]. Each concentration was set to 0.3 ng/mL, the average hormone concentration in non-pregnant women, and 17 ng/mL, the highest of the mean values of 2–17 ng/mL of plasma concentrations of E2 in the umbilical vein, and 7 ng/mL of the mean values of 2–7 ng/mL in the umbilical artery reported at 28–40 weeks’ gestation [26,27,28,29,30]. The reason for choosing the highest concentration In each range was to clearly see the difference in melanin expression due to the difference in hormone concentrations compared to non-pregnant women.

#### 3.2.1. Melanin Expression in Co-Cultured HDF and HEM in 2D

As a comparison group to confirm the functionality of the developed Artificial Skin Platform, HDF and HEM were co-cultured in 2D, with oxygen concentrations of 2.5–3% and 21%, and E2 concentrations of 0.3, 7, and 17 ng/mL, respectively, and the resulting melanin expression was analyzed. At this time, we checked whether the melanin expression trends were similar to the melanin expression mechanism in actual pregnant women’s skin. This confirmed that cells cultured in 2D can simulate the pigmentation of pregnant women’s skin.

**Figure 5:**
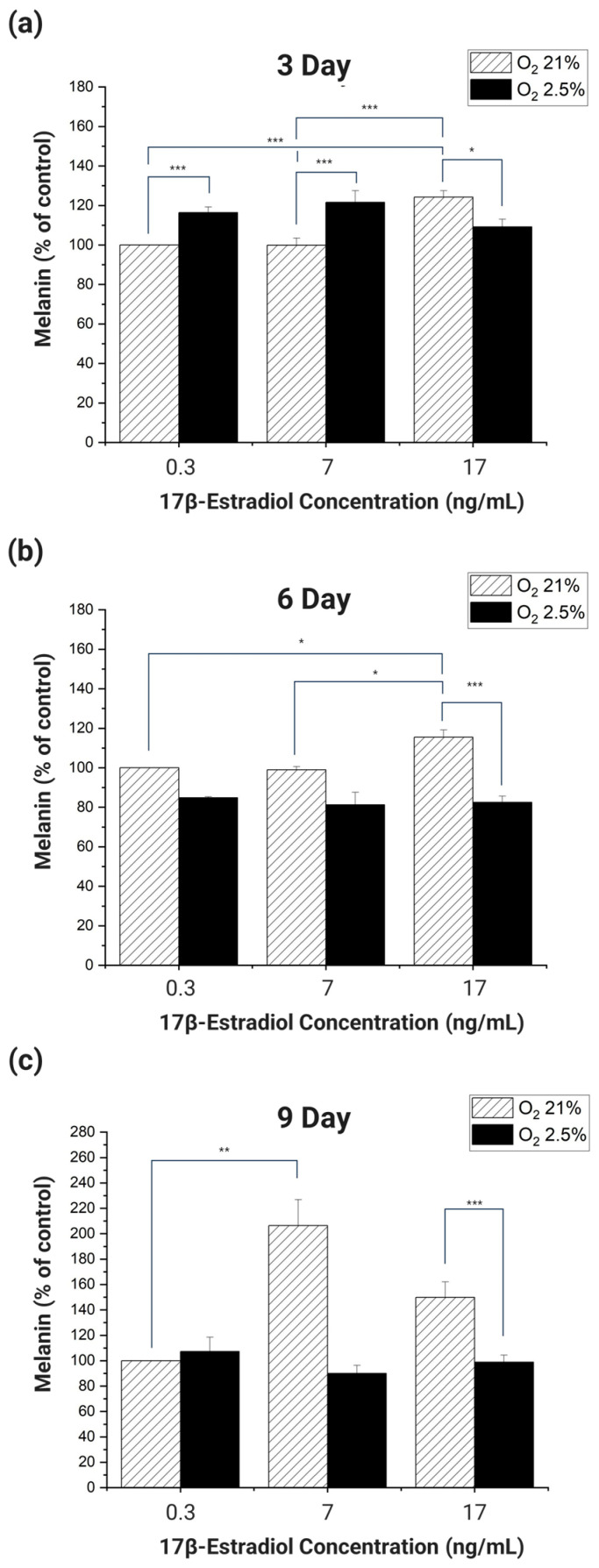
Results of melanin expression analysis in response to oxygen concentration and E2 concentration in 2D cultures of HDF and HEM. The control of the experiment was set to 21% oxygen concentration and 0.3 ng/mL E2 concentration as the value of non-pregnant women. HDF and HEM were co-cultured with an oxygen concentration of 21% and 2.5–3% and E2 concentration of 0.3, 7, 17 ng/mL for (**a**) 3 days, (**b**) 6 days, (**c**) 9 days and analyzed for melanin expression. There were differences in melanin production in response to oxygen and E2 concentrations, but no consistent trends were identified. (The *p* values were calculated by ANOVA. (*n* = 6; *, *p* < 0.05; **, *p* < 0.01; ***, *p* < 0.001).

If the trend of melanin expression on each day of culture is the same as the trend of melanin expression in real pregnant women when the E2 and oxygen concentrations are set according to the experimental conditions, it can be said that the cells cultured in 2D mimic the pigmentation trend of real pregnant women. Therefore, we divided the culture days into 3 days, 6 days, and 9 days and analyzed whether the melanin expression trend of the cultured cells on each day was similar to the melanin expression trend of real pregnant women (Figure 5).

When HDF and HEM were co-cultured for 3 days and the melanin produced was analyzed, the melanin produced by cells cultured in 21% oxygen increased in proportion to the increase in E2 concentration (Figure 5a). Among the experimental groups cultured in 21% oxygen, cells cultured at E2 concentrations of 0.3 ng/mL and 7 ng/mL produced significantly more melanin than cells cultured at 17 ng/mL (*p* < 0.001). Cells cultured in 2.5–3% oxygen had the highest melanin at an E2 concentration of 7 ng/mL and the lowest at 17 ng/mL. In addition, melanin significantly increased with decreasing oxygen concentration in cells cultured at E2 concentrations of 0.3 ng/mL and 7 ng/mL (*p* < 0.001). Cells cultured in 2.5–3% oxygen had the highest melanin at an E2 concentration of 7 ng/mL and the lowest at 17 ng/mL. In addition, cells cultured in 0.3 ng/mL and 7 ng/mL of E2 significantly increased melanin with decreasing oxygen (*p* < 0.001). However, at an E2 concentration of 17 ng/mL, melanin production decreased significantly with decreasing oxygen concentration (*p* < 0.05).

When HDF and HEM were co-cultured for 6 days and melanin was analyzed, cells cultured in 21% oxygen showed a proportional increase in melanin in response to E2 (Figure 5b). However, cells cultured at 2.5–3% oxygen produced less melanin overall than cells cultured at 21% oxygen. Among the groups cultured in 21% oxygen, each group cultured at E2 concentrations of 0.3 ng/mL, 7 ng/mL had a significant increase in melanin compared to the group cultured at 17 ng/mL (*p* < 0.001). This was the same result as in the 3-day incubation. Of the groups incubated at 2.5–3% oxygen, only the group incubated at 17 ng/mL of E2 had a significant increase in melanin compared to the group incubated at 21% oxygen and 17 ng/mL of E2 (*p* < 0.001).

When HDF and HEM were co-cultured for 9 days and melanin was analyzed, cells cultured at 2.5–3% oxygen showed no significant changes in response to E2 concentration, but overall, there was a significant difference in expression compared to cells cultured at 21% oxygen (Figure 5c). In cells cultured in 21% oxygen, there was a significant increase in melanin proportional to E2 concentration between cells cultured at 0.3 ng/mL and 7 ng/mL (*p* < 0.01). In addition, melanin significantly decreased with decreasing oxygen concentration in cells cultured at an E2 concentration of 17 ng/mL (*p* < 0.001).

The overall analysis of the data from the experiments showed that the cells cultured in the 2D culture environment did not produce melanin that was proportional to the E2 concentration and inversely proportional to the oxygen concentration, even when the E2 concentration and oxygen concentration were applied to be similar to the environment in the body of a pregnant woman. These results confirm that cells cultured in a 2D culture environment are unable to mimic the melanogenic response to O_2_ and E2 concentrations in the skin of real pregnant women [27,28,29,30,45,46].

#### 3.2.2. RT-PCR Analysis of Melanin Expression-Related Genes in HDF and HEM Co-Cultured in 2D

To analyze by RT-PCR how the expression of melanin-related genes in HDF and HEM co-cultured in 2D changes in response to the oxygen and E2 concentrations applied to the cell culture to simulate the environment in the body of a pregnant woman, we selected genes that affect melanin synthesis. For this experiment, we chose TYR, TYRP1, and MITF as target genes. TYR and TYRP1 are essential proteins for melanin biosynthesis. Their functions and activities are still not fully understood, but TYR is relatively well established, while TYRP1 is still a mystery in terms of catalytic activity [50]. MITF is involved in the development, proliferation, and survival of melanocytes and is an essential regulator of the expression of enzymes and structural proteins involved in melanogenesis [51]. We used β-actin as a reference gene for TYR, TYRP1, and MITF, the genes selected for RT-PCR analysis, and analyzed the changes in expression by RT-PCR as a function of oxygen concentration and E2 concentration [52,53,54].

**Figure 6:**
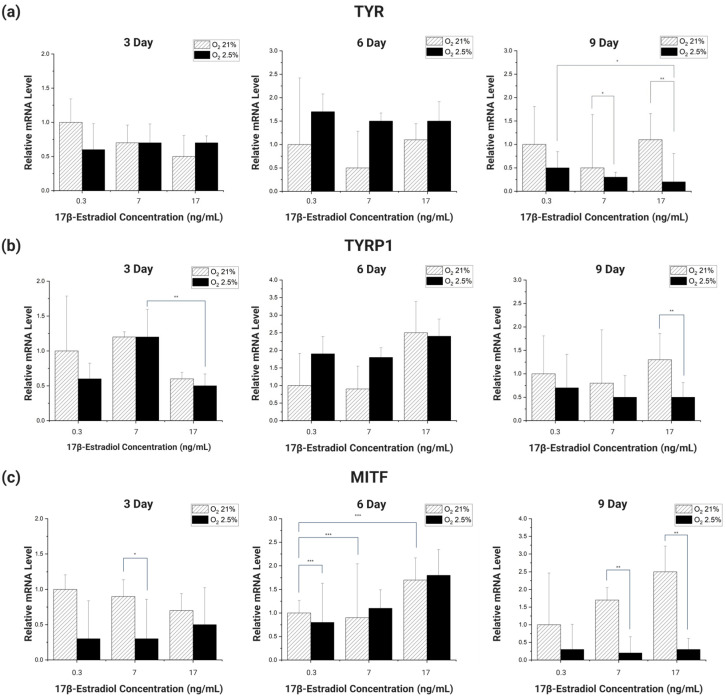
Expression of melanin-related genes was analyzed by RT-PCR at 3, 6, and 9 days after co-culture of HDF and HEM in a 2D culture environment, with oxygen and E2 concentrations adapted to the experimental conditions. Data are normalized for β-actin mRNA levels and are expressed relative to controls. Control was set as cells grown in a culture environment with an oxygen concentration of 21% and an E2 concentration of 0.3 ng/mL. (**a**) Expression of TYR at 3, 6, and 9 days, according to oxygen concentration and E2 concentration. (**b**) Expression of TYRP1 at 3, 6, and 9 days, in response to oxygen and E2 concentrations. (**c**) Expression of MITF at 3, 6, and 9 days, in response to oxygen and E2 concentrations. *p* values were calculated by ANOVA. (*n* = 3; *, *p* < 0.1; **, *p* < 0.05; ***, *p* < 0.01).

Looking at the overall expression trend of the genes (Figure 6), the gene expression was initially high in the 21% oxygen environment, but as the incubation period reached 6 days, the gene expression of the cells cultured in the 2.5–3% oxygen environment improved rapidly. However, as the incubation period increased, the gene expression of the cells cultured in the 2.5–3% oxygen environment decreased sharply, increasing the gene expression difference with the cells cultured in the 21% oxygen environment.

Different types of genes had different incubation periods in which they showed significant trends. For TYR, there was no significant trend at 3 and 6 days of incubation, but at 9 days, there was a significant decrease in the gene expression trend of cells cultured in an environment with 2.5–3% oxygen and 0.3 ng/mL E2, as well as the same oxygen and E2 concentration of 17 ng/mL (*p* < 0.1). In addition, in cells cultured in different oxygen concentrations of E2 concentration 7 ng/mL, 17 ng/mL, the expression of genes decreased significantly with decreasing oxygen concentration (*p* < 0.1, *p* < 0.05) (Figure 6a).

When TYRP1 was incubated for 3 days, there was a significant decrease in E2 concentration between 7 ng/mL and 17 ng/mL in the 2.5–3% oxygen group (*p* < 0.05). In addition, on day 9, the expression of the gene was significantly reduced with decreasing oxygen concentration in the experimental group with E2 concentration of 17 ng/mL (*p* < 0.05) (Figure 6b).

MITF did not show a significant trend in gene expression when incubated for 3 days. However, a significant trend was seen after 6 days of incubation. At 6 days of incubation, compared to the 21% oxygen concentration and 0.3 ng/mL E2 concentration, there was a slight decrease in gene expression at the same oxygen concentration and 7 ng/mL E2 concentration, and a significant increase at 17 ng/mL E2 concentration (*p* < 0.01). Also, when the incubation period reached 9 days, the decrease in gene expression with decreasing oxygen concentration was significant between the different oxygen concentrations of E2 concentration 7 ng/mL and E2 17 ng/mL (*p* < 0.05) (Figure 6c).

The RT-PCR analysis of the genes showed that TYR and MITF showed significant trends in the expression of the genes at specific incubation periods. The difference is that TYR showed a significant trend of decreasing gene expression with decreasing oxygen concentration, while MITF showed a significant trend of increasing gene expression proportionally with E2 concentration. These results lead us to speculate that the factors affecting the expression of each gene are different depending on its type. These results also lead us to conclude that there is no clear trend to correlate melanin changes with the changing body environment of pregnant women [27,28,29,30,45,46].

### 3.3. Pigmentation of 3D Artificial Skin on a Skin-on-a-Chip Based on Simulation of a Pregnant Woman’s Body Environment

Histological analysis methods H & E and Fontana Mason staining were performed to analyze the melanin trends of 3D artificial skin cultured in dynamic culture on the Artificial Skin Platform in response to oxygen and E2 concentrations, conditions that mimic the environment of a pregnant woman’s body. In this process, the cells seeded with collagen type I were not directly in contact with the dynamic flow due to the fibrin layer at the bottom, so the effects of dynamic flow on the cells were not considered.

**Figure 7:**
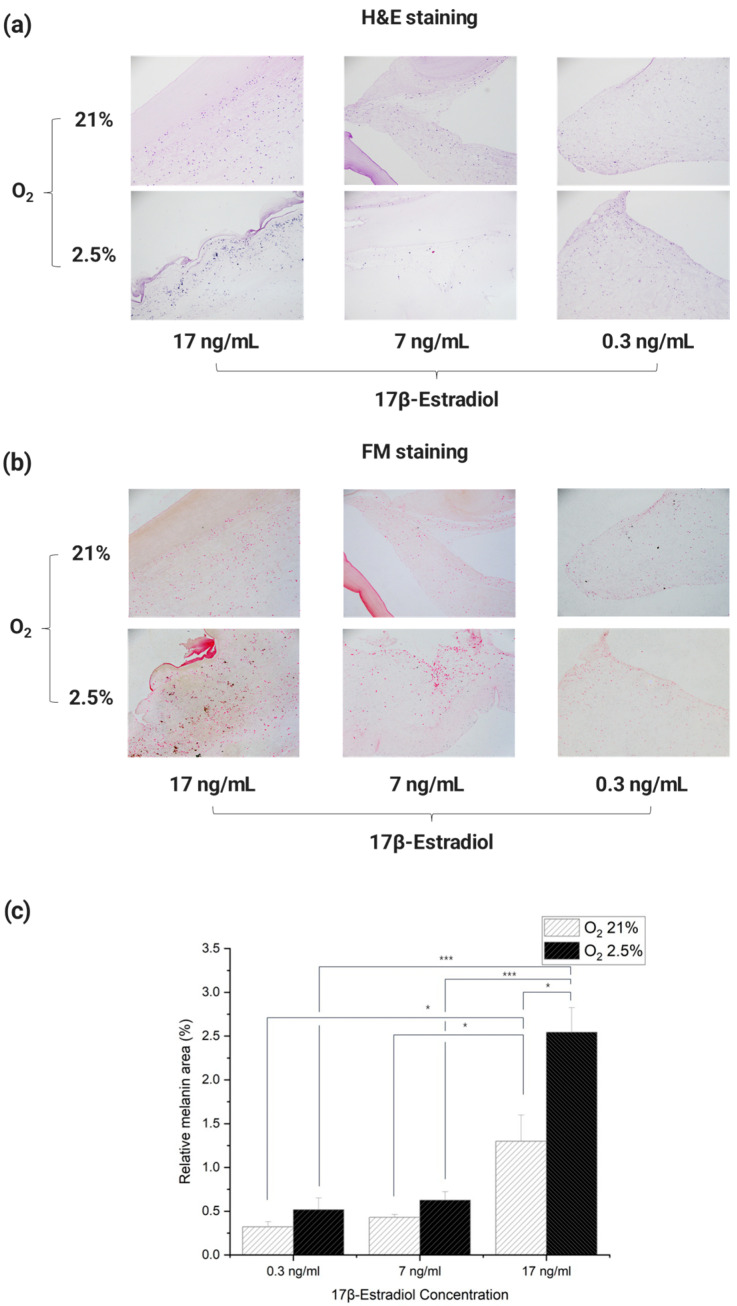
Results of H & E and Fontana Mason staining of cut sections of 3D artificial skin. (**a**) H & E staining results, according to oxygen concentration and E2 concentration, in artificial 3D skin culture. (**b**) Fontana Mason staining results, according to oxygen concentration and E2 concentration in 3D artificial skin culture. (**c**) The ratio of melanin pigment to the cross-sectional area of 3D artificial skin by Fontana Mason staining results. The *p*-value was calculated by ANOVA. (*n* = 3; *, *p* < 0.05; ***, *p* < 0.001).

The results of staining each Incubated 3D artificial skin with H & E showed that cytoplasm and cell nuclei were observed in each cross-section (Figure 7a). Images of each section stained with Fontana Mason were used as Image J to determine the percentage of melanin pigment in the total cross-sectional area. For each culture, paraffin blocks were cut three times, the obtained cross sections were stained with Fontana Mason, and the process of analysis was repeated to quantify the values (Figure 7b,c).

The results showed that melanin expression tended to increase in an environment similar to that of a pregnant woman’s body. In particular, the results showed that melanin expression tended to increase at E2 concentrations in the third trimester of pregnancy. The lowest cross-sectional area-to-melanin ratio was observed in the culture environment similar to that of non-pregnant women, with an oxygen concentration of 21% and an E2 concentration of 0.3 ng/mL. In addition, the difference in cross-sectional area-to-melanin ratio between 21% oxygen and 2.5–3% in culture with E2 concentrations of 0.3 and 7 ng/mL was similar, with a difference of approximately 1.6 and 1.5 times, respectively. However, in the culture environment with an E2 concentration of 17 ng/mL, the difference in cross-sectional area-to-melanin ratio was about 2.0 times greater than in the other experimental groups, depending on the difference in oxygen concentration. This confirms that the difference in melanin expression by E2 concentration is greater than the difference in melanin expression by oxygen concentration [45,46,55]. It is also speculated that the expression of melanin does not simply increase linearly with E2 concentration, but that there is a sharp rise in the amount of melanin beyond a certain E2 concentration threshold.

The results of the statistical analysis showed that there were no significant differences between the groups with the same E2 concentration and different oxygen concentrations, except for the group with E2 concentration of 17 ng/mL. In addition, each group had a significant trend with the group with E2 concentration of 17 ng/mL. The experimental group with 21% oxygen and 0.3 ng/mL E2 concentration had a significant increase in melanin compared to the experimental group with the same oxygen and 17 ng/mL E2 concentration (*p* < 0.05). There was also a significant increase in melanin between the 21% oxygen and 7 ng/mL E2 group and the same oxygen and 17 ng/mL E2 group (*p* < 0.05). Melanin increased significantly (*p* < 0.001) between the 2.5–3% oxygen and 0.3 ng/mL E2 concentration group and the same oxygen, 17 ng/mL E2 concentration group. There was a significant increase in melanin between the 2.5–3% oxygen and 7 ng/mL E2 concentrations and the same oxygen and 17 ng/mL E2 concentrations (*p* < 0.001).

Taken together, these results show that just as the incidence of skin pigmentation in real pregnant women increases rapidly in the third trimester of pregnancy, melanin increased rapidly in the 3D artificial skin with the E2 concentration applied at that time. In other words, the tendency of melanin expression in the 3D artificial skin was similar to that of pregnant women. In addition, the significant data obtained from the ANOVA analysis showed that the degree of melanin expression was much more affected by the difference in E2 concentration than by the difference in oxygen concentration. This is consistent with the increase in pigmentation cases in pregnant women in proportion to their E2 levels, which rise sharply in the third trimester of pregnancy [26,27,28,29,30,56]. Furthermore, the melanin levels at E2 concentrations of 17 ng/mL and 7 ng/mL are the E2 concentrations found in the umbilical vein and umbilical artery, respectively, which were set for detailed comparison in this study. When comparing the amount of melanin expression at the two concentrations, the amount of melanin expressed in the umbilical vein condition was much higher. Therefore, this result suggested that plasma flowing from the umbilical vein is a more influential factor in pigmentation. In other words, the results obtained through the 3D artificial skin implemented through the Artificial Skin Platform tended to be similar to the actual skin of pregnant women, so it can be used as a platform for treating skin pigmentation in pregnant women.

## 4. Conclusions

In this study, we implemented a 3D Artificial Skin Platform that can be used to identify the causes of skin hyperpigmentation in pregnant women and develop treatments. We showed that the cells cultured in this platform have a greater similarity to the melanin expression trend of actual pregnant women than the cells cultured in the 2D model. Therefore, we have verified that a 3D platform that is more similar to the actual in vivo is a better platform to implement a pigmentation model that occurs in the body of a pregnant woman compared to the existing 2D culture platform.

A variety of microfluidic-based 3D skin chips have been developed and validated that can be used to conduct research by implementing artificial skin [57,58]. These 3D skin research platforms have been applied in a wide range of fields such as drug toxicity, efficacy testing, wound healing, skin aging, infection, etc. through more advanced skin simulation. Further extensions are being made to advanced artificial skin systems that mimic the function of the skin through integration with sensor modules [59,60,61]. In comparison, the pigmentation analysis platform for pregnancy developed in this study can be considered a rather simplified platform, as it is implemented with only two types of cells rather than simulating all the layers of the actual skin. However, we obtained significant results (compared to the 2D platform) that quickly validated the utility of this 3D skin platform. Therefore, in the future, it is expected that the platform will be improved by adding more diverse skin cells, especially keratinocytes, to increase the biomimicry.

In this context, the 3D Artificial Skin Platform developed in this study is expected to be useful as a model of skin pigmentation in pregnant women and as a platform for identifying the mechanisms of skin pigmentation in pregnant women and developing therapies.

## Figures and Tables

**Table 1:** Primers used for RT-PCR.

Gene	Primer Sequence of PCR (5′-3′)
Beta-actin	F: AAG GTG ACA GCA GTC GGT T
	R: TGT GTG GAC TTG GGA GAG G
TYRP1	F: TCT CTG GGC TGT ATC TTC TTC C
	R: GTC TGG GCA ACA CAT ACC ACT
TYR	F: CAA TGT CCC AGG TAC AGG GAT
	R: GTA GGA TTC CCG GTT ATG TCC A
MITF	F: GGG AGC TCA CAG CGT GTA TT
	R: ATG GTT CCC TTG TTC CAG CG

## Data Availability

Data are contained within the article.
